# Data-driven approaches for predictive modeling of N77/N78 band 5G microstrip patch array antenna using curve fitting and supervised regression

**DOI:** 10.1038/s41598-026-58474-2

**Published:** 2026-06-27

**Authors:** Md. Ashraful Haque, Dipon Saha, Md Afzalur Rahman, Md Sultan Mahmud, Jun-Jiat Tiang, Narinderjit Singh Sawaran Singh, Ruwaybih Alsulami, Saeed Alzahrani

**Affiliations:** 1 Department of Electrical and Electronic Engineering, University of Liberal Arts Bangladesh (ULAB), Dhaka, 1207 Bangladesh; 2 Department of Electrical and Electronic Engineering, Daffodil International University, Dhaka, 1216 Bangladesh; 3 Department of Electrical and Electronic Engineering, University of Asia Pacific, 74/A, Green Road, Farmgate, Dhaka, 1205 Bangladesh; 4 Centre for Wireless Technology, CoE for Intelligent Network, Faculty of Artificial Intelligence & Engineering, Multimedia University, Persiaran Multimedia, 63100 Cyberjaya, Selangor Malaysia; 5 Faculty of Data Science and Information Technology, INTI International University, Persiaran Perdana BBN, Putra Nilai, 71800 Nilai, Negeri Sembilan Malaysia; 6 Department of Electrical Engineering, Umm Al-Qura University, Makkah, Saudi Arabia; 7 Department of Electrical Engineering, University of Tabuk, Tabuk, Saudi Arabia

**Keywords:** Antenna, Curve fitting, Machine-learning, CST, ADS, Industrial and innovation, Engineering, Materials science, Mathematics and computing

## Abstract

This paper deals with the design of a single-element and *1× 2* bandwidth microstrip patch array antennas for N77/N78 band applications. The presented antennas are designed on a low-cost FR4 epoxy substrate with a dielectric constant of 4.3 and a thickness of 1.6mm while considering manufacturability issues. In order to evaluate the antenna’s characteristics, an equivalent circuit model of the proposed microstrip patch array will be built in Keysight ADS, and the reflection coefficient will be compared with the simulated result obtained with CST Studio Suite. An implemented prototype is also measured for the validity of the simulated results, and good agreement between the measurement and simulation is obtained. Besides the classical electromagnetic simulation, supervised machine learning (ML) algorithms are used to estimate the main antenna parameters, such as the bandwidth and the center resonant frequency of the investigated antenna. 203 simulation-based data samples are generated by using CST Microwave Studio and employed to train five regression models, including GB regression, ET regression, DT regression, RF regression, and XGB regression. The models are evaluated in terms of variance score, coefficient of determination (R$^2$), mean absolute error (MAE), mean square error (MSE), and root mean square error (RMSE). Among all tested models, the Random Forest regression model produces the best results in terms of error and accuracy for predicting both bandwidth and center resonant frequency. The proposed work concludes as an effective antenna design method through integration of full-wave simulation, measurement, equivalent circuit modeling, and machine learning-based prediction for robust N77/N78 band implementation.

## Introduction

The n77/n78 5G band is essential to the growth of 5G networks because it provides a mid-band spectrum that achieves a good mix between coverage and capacity, thereby allowing for a diverse set of applications and services for both consumers and businesses^1^. Transmission and reception efficiency is maximized in the n77 and n78 5G channels since antennas optimized for these bands are often built to cover only those frequencies. The n77 band antennas should be operated at the frequency of 3.7 GHz while the n78 band antennas should be working at a resonant frequency of 3.5 GHz.^2^. The below-6 GHz frequencies, or the sub-6 GHz band, are core to the 5G spectrum and offer their own advantages and applications.^3^. To understand why the n77 and n78 bands are considered very strategic, we need to understand a little about the broader capabilities and aspirations of 5G as a communications standard.

5G, “Fifth generation cellular” is the most recent and cutting-edge iteration of mobile communication standards. It follows versions such as 1G (analog cellular), 2G (limited digital cellular), 3G (mobile data’s debut) and 4G (high-speed data and enhanced multimedia capabilities)^4^. 5G’s benefits include increased throughput, decreased latency, greater connection, more flexible use cases, and the ability to slice networks. Theoretical transfer rates for 5G networks can reach gigabits per second. These fast transfer rates are an essential part of 5G, allowing for a plethora of services and apps that can make use of the improved wireless connectivity^5^. Fifth-generation cellular networks enable low-power Internet of Things (IoT) applications. The unique characteristics of 5G make it ideal for connecting a multitude of IoT devices with diverse power needs.^6^. Delivering these capabilities requires antenna systems that are specifically engineered to operate efficiently within the designated 5G bands, making the choice of antenna topology a central design consideration.

A microstrip patch antenna is a trim, simple antenna configuration. Because of its simple design, high-frequency selectivity, and adaptability, it is widely employed in wireless communication systems^7^. Improved performance features, including gain, directivity, and beam steering, can be attained by configuring numerous microstrip patch antennas in an array. Applications that demand improved wireless communication, radar systems, and other advanced technologies often use such arrays. An antenna is said to be of the type known as a microstrip array antenna if it employs the use of microstrip patch components that are organized in an array configuration on a dielectric substrate. The capacity to generate a variety of radiation patterns and beam steering capabilities are two of the many benefits that come with using an antenna of this sort. Other benefits include the antenna’s low profile and ease of construction^8^. Beyond the physical configuration of the antenna elements, the feed mechanism and simulation port excitation method are equally critical factors that govern impedance matching and overall antenna performance.

The microstrip patch antenna performance is strongly related to the feeding technique and port excitation. Popular feeding techniques are inset microstrip line feed, coaxial probe feed, aperture-coupled feed, and proximity-coupled feed. These techniques have different pros and cons in terms of impedance matching, bandwidth, fabrication complexity, and undesired radiation. The inset feed provides a straightforward approach for impedance matching by varying the depth of the feed notch into the patch without requiring additional layers or via holes. Port excitation is also very important to correctly predict an antenna’s performance in full-wave EM simulation. Quasi-TEM mode excitation by a waveguide port that is defined at the cross-sectional plane of the 50 *Ω* microstrip feed line at the edge of the substrate, where both the microstrip feed line width and the substrate thickness are considered in the determination of the reflection coefficient (S11). A single waveguide port at the input of the microstrip power divider network is sufficient to excite both radiating elements in a *1× 2* array configuration evenly. This makes it possible to analyse the radiation pattern and impedance matching of the array accurately over the full frequency of interest. The feedline-compatible underlying substrate material, in conjunction with the feed design, plays an important role in controlling the resonant response, the efficiency and the practical feasibility of fabricating the antenna.

The selection of substrate material has a significant effect on the antenna’s electric size, radiation efficiency and potential bandwidth. Because of its low price, wide commercial availability, and good compatibility with traditional printed circuit board (PCB) fabrication processes, the FR4 epoxy ($\varepsilon _r$ = 4.3, loss tangent tan $\delta \approx$ 0.02) is the most widely used substrate material for microstrip antenna design and related prototyping. While FR4 possesses a significantly higher dielectric loss than specialised microwave laminates such as Rogers RT/duroid, its average dielectric constant of $\varepsilon _r$ = 4.3 provides a feasible/compact antenna size for operation within the 3.5-3.7 GHz operational band of the n77 and n78 bands, thus rendering it a viable and cost-effective substrate option for the proposed design. In order to compensate for the relatively high loss tangent of FR4 and to achieve reasonable radiation efficiency and input impedance bandwidth, a novel optimisation procedure of the patch dimensions, feeding structure and ground plane configuration is presented. As well as the feed structure, the substrate material is a crucial element which affects the resonance characteristic, efficiency and feasibility of an antenna. Along with substrate selection and structural optimization, data-driven modelling has also become an important approach for improving antenna design and performance prediction.

In recent studies, several research works that have utilized machine learning methods for parameter prediction and modeling of the antenna. The research^9^ considered the application of supervised machine learning methodologies to the estimation of printed antenna geometric and electrical parameters. On the same note,^10^ studied the decision tree based forward and inverse modeling for the design of microstrip antenna whereas in^11^ a ML based hybrid EM modeling technique for UWB MIMO antennas is presented. These study illustrates the increasing importance of ML in antenna engineering, mainly to decrease the design complexity and increase the prediction accuracy. Since ML models have the ability to learn nonlinear relationships between antenna dimensions and performance parameters, they allow for more rapid estimation of resonant frequency, bandwidth, gain, and similar features. Hence, applying ML is a time-efficient complementary approach to the traditional antenna design based on simulations.

With the competitive RF design and the more rigorous analysis technique, the proposed antenna, whose geometry is tabulated in Table 6, provides a better overall compromise than the cited references. It is based on FR4 substrate ($\varepsilon _r$= 4.3) at frequencies 3.5 and 3.7 GHz with reflection coefficients of -40.5 and -38.3 dB, radiation efficiency = 85%, Maximum Gain = 5.1 dB and PCB size of 0.58$\lambda _\circ \times$0.93$\lambda _\circ$. The proposed antenna achieves higher gain while still maintaining good efficiency when compared with low-gain configurations such as^12^ (1.58 dB),^13^ (3.55 dB),^14^ (0.53 dB), and^15^ (3.36 dB). Moreover, it demonstrates a remarkable improvement in efficiency 85%. which implying a higher gain-efficiency point of balance. Furthermore, it stands out among all the considered lines since it is the only design in the table that uses both RLC modelling and ML processing.

## Research gap and contributions

Due to the rapid development of sub-6 GHz 5G systems, compact, high-gain antennas for the n77 and n78 bands are in demand. Microstrip Patch (MSP) antennas have also been investigated in the past in conjunction with ML-based optimisation and RLC equivalent circuit modelling, although these approaches are typically employed individually or to a limited extent. Hence, in terms of compactness, integrated antenna solutions are not available that can simultaneously support dual-use applications with dual-resonances, high-gain, high-efficiency, reliable bandwidth, and circuit-level interpretability in a monolithic design flow. In existing techniques, a performance metric is generally improved at the cost of other performance metrics, such as high gain at low efficiency, or proper impedance matching without predictive insight. In addition, RLC modelling is sometimes introduced without a clear relationship to physical shape or resonance characteristics, and ML is often employed as a post-design predictor. This gap underlines the importance of a detailed design procedure for compact multi-application antennas based on a comprehensive application of geometry, simulation, measurement, RLC modelling, and data-driven prediction. The work presented in this paper proposes a unified design and analysis methodology for a single-element and *1× 2* array microstrip patch antenna for dual applications in N77 and N78 channels to bridge this shortcoming. To have a reliable, understandable and high-performing antenna design, the method combines full-wave analysis, circuit-level modelling, measurement verification and supervised regression-based prediction. The following are the main contributions of this work:

- Compact antenna design: A compact single layer single element rectangular microstrip patch antenna is presented for wideband applications by employing FR4-substrate ($\varepsilon _r$=4.3) with 1.6 mm thickness. The full size of the antenna is 0.58$\lambda _\circ \times$0.93$\lambda _\circ$ and is designed based on *1× 2* array configuration for use in space constrained wireless environment.
- Enhance performance: Peak gain 5.1dB and radiation efficiency 85% with dual resonances at 3.5 GHz and 3.7 GHz. It can be used for dual applications in N77 and N78 channels, which demonstrates a small and efficient solution for sub-6 GHz 5G applications.
- RLC Equivalent Circuit Modeling: An RLC equivalent circuit in ADS is developed to verify the full-wave simulations, to represent antenna response precisely and to provide a circuit level insight into resonance nature.
- Supervised regression-based prediction: Applied five regression models to predict resonance frequency and bandwidth, with Random Forest Regression achieving the best performance:
  - Resonance: MAE = 0.5621%, MSE = 0.0154%, RMSE = 1.2402%, R$^2$ = 99.20%, Variance score = 99.20%.
  - Bandwidth: MAE = 0.4801%, MSE = 0.0057%, RMSE = 0.7540%, R$^2$ = 99.38%, Variance score = 99.40%.
- Antenna fabrication and measurement validation: The antenna was prototyped and tested to verify the performance. The measured results agreed very well with the simulation results, which verify the proposed design is reliable, accurate and that it is practically feasible. Dual-application array operation: Demonstrated that the *1× 2* array configuration effectively supports dual applications in both N77 and N78 channels, ensuring practical utility in modern sub-6 GHz 5G systems.

With the antenna design being well-balanced, dual applications, high gain and efficiency, accurate predictive modeling and circuit-level interpretable this solution is a good candidate for 5G sub-6 GHz deployments.

## Antenna design and configuration

**Fig. 1:**
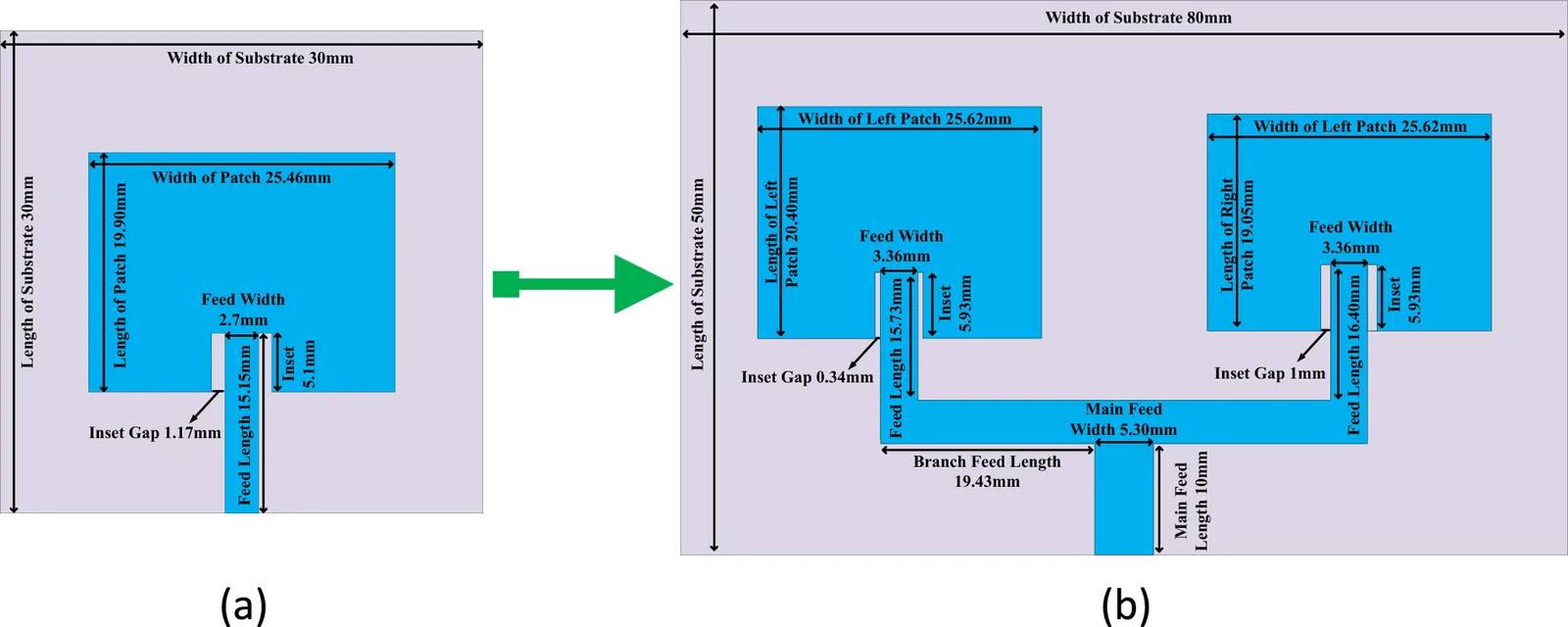
The Geometry of Antenna. (**a**) Single element. (**b**) Array antenna.

The CST MW software package developed by Computer Simulation Technology (CST) was employed to conduct simulations of the operational characteristics of the N77 and N78 band application antenna.

The dimensions of the proposed microstrip antenna were initially calculated using conventional transmission-line model equations before optimizing through parametric analysis. The resonant frequency of a rectangular microstrip antenna mainly depends on the patch length, width, substrate dielectric constant, and substrate thickness.

The above design equations are employed to find the initial dimensions of the mentioned microstrip patch antenna. In particular, the patch width is determined by Eq. (1) and the patch length is derived from Eqs. (2), (3), (4) and (5)^16^.

1 $$\begin{aligned} W = \frac{c_\circ }{2 f_r}\sqrt{\frac{2}{\varepsilon _r + 1}} \end{aligned}$$

2 $$\begin{aligned} L = L_{eff} - 2 \Delta L \end{aligned}$$

3 $$\begin{aligned} L_{eff} = \frac{c_\circ }{2 f_r \sqrt{\varepsilon _{r~eff}}} \end{aligned}$$

4 $$\begin{aligned} \Delta L = 0.412 \left[ \frac{(\varepsilon _{r~eff} + 0.3)(\frac{W}{h}+0.264)}{(\varepsilon _{r~eff} - 0.3)(\frac{W}{h} + 0.813)} \right] \end{aligned}$$

5 $$\begin{aligned} \varepsilon _{r~eff} = \frac{\varepsilon _r + 1}{2} + \frac{\varepsilon _r - 1}{2} \sqrt{1 + 12 \frac{h}{W}} \end{aligned}$$

Where,

$$\begin{aligned} c_\circ&= \text {Electromegnetic wave velocity} \\ f_r&= \text {Lowest resonance frequency} \\ \varepsilon _r&= \text {Dielectric constant of substrate} \\ L_{eff}&= \text {Effective length} \\ \Delta&= \text {Length extension} \\ \varepsilon _{r~eff}&= \text {Effective dielectric constant} \end{aligned}$$

FR-4 is the substrate material for the antenna, where the dielectric constant is equal to $\varepsilon _r$ = 4.3 and the height is 1.6 mm. The substrate is covered with a copper layer of thickness 0.035 mm. These substrate characteristics and a desired resonant frequency of 3.5GHz are used to derive the initial values for the antenna’s length and width. The antenna is simulated with these approximate parameters and further optimized by a parametric study in Parametric analysis sect. The final design of the proposed microstrip antenna is depicted in Fig. 1, where Fig. 1 (a) is the single-element patch antenna and Fig. 1b is the array antenna configuration.

The microstrip feed-line width was calculated to maintain a characteristic impedance of 50 *Ω*. The feed-line dimensions were further optimized during simulation to improve impedance matching and minimize reflection coefficient. The optimized feed-line width was selected as 3.36 mm for 50 *Ω* matching. To achieve proper impedance matching, an inset-feed technique has been employed. The inset gap position was optimized to match 50 *Ω* input impedance at the operating frequency of 3.5 GHz, and presented in Fig. 1. The inset feed shifts the feeding point toward the patch center, where the input impedance decreases from its maximum edge value. The antenna array feeding network utilizes a microstrip power divider structure to distribute power uniformly among the radiating elements. Quarter-wave impedance transformation principles were considered during the feed network design. The feed network dimensions were optimized to ensure equal power distribution and minimize transmission losses, and presented in Fig. 1. The ground plane is located on the back of the dielectric substrate. The width of the substrate and the length of the substrate are both 30 millimeters (mm) for the single element design. The substrates have dimensions of 80 mm in width, and 50 mm in length for the array design. The detailed dimension of each components of the antenna has been presented in the Fig. 1.

## Parametric analysis

**Fig. 2:**
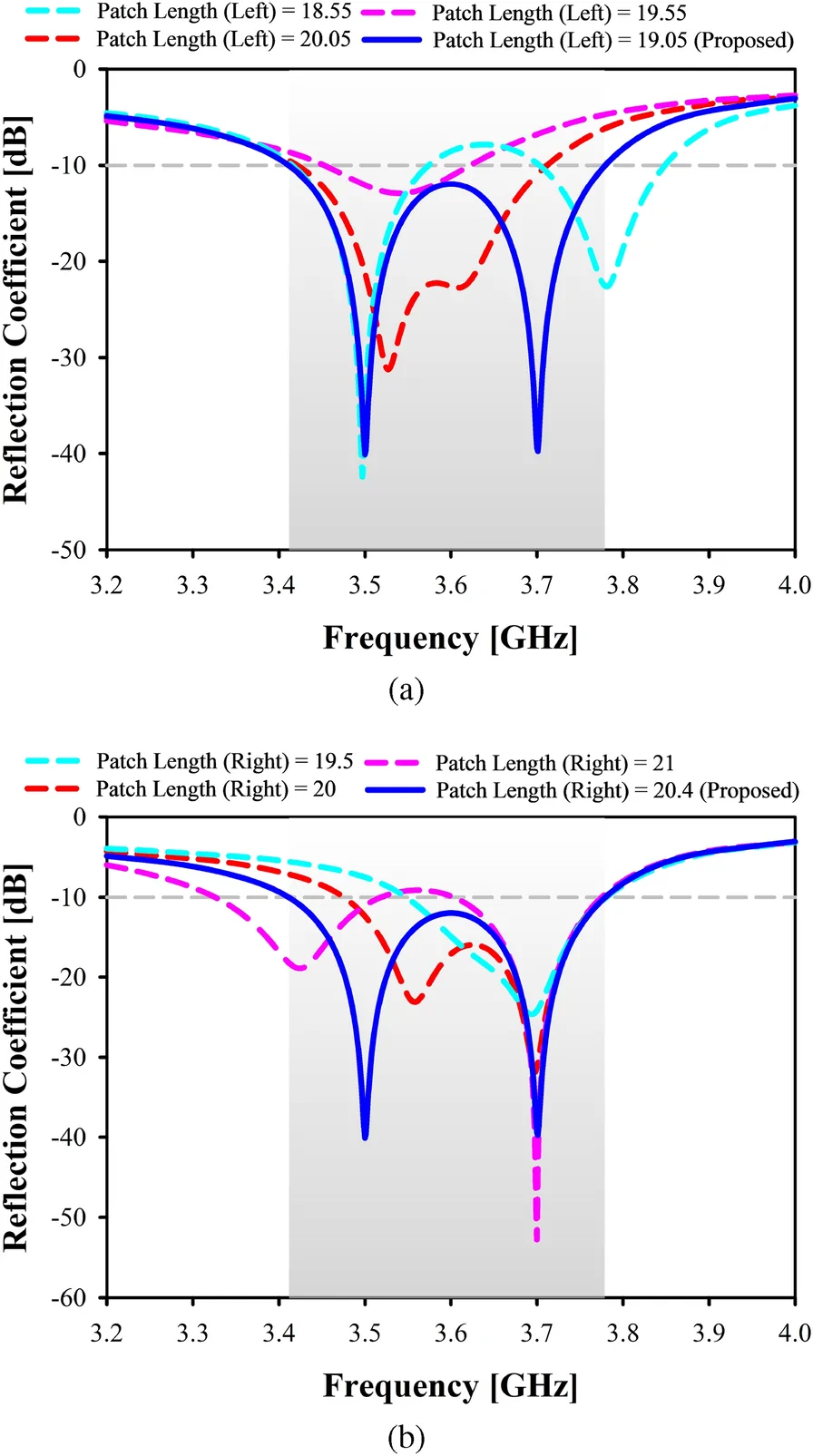
Simulated reflection coefficient for different lengths of Patch. (**a**) Left Patch. (**b**) Right Patch.

This part considers the impact of various factors, such as the size of the Patch along the length and the width or spacing of the Inset. Since the antenna consists of two patches, this analysis should be carried out for each patch. The length of the patch, which should be closer to a fourth of lambda, is a primary factor in determining the resonance frequency of patch antennas. As can be seen in Fig. 2a and b, the PL and PLR values that were decided upon for this antenna produce the best possible results.

**Fig. 3:**
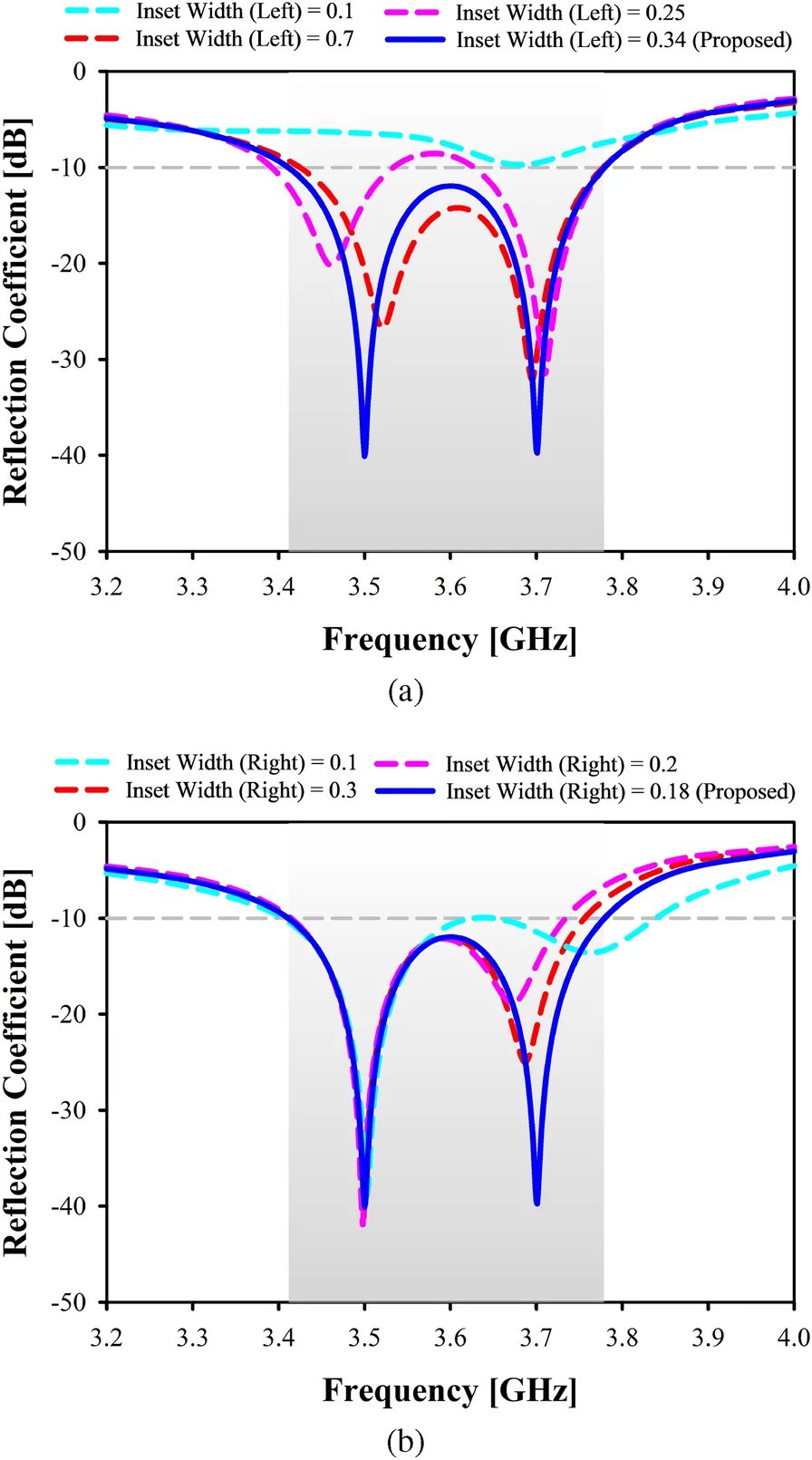
Simulated reflection coefficient for different widths of Inset Gap. (**a**) Left Inset. (**b**) Right Inset.

Impedance matching between the antenna and the feed line can be achieved through an inset gap. In this case, the inset widths were varied throughout a wide range of values; nonetheless, the analysis reveals that the values chosen for IW and IWR are optimal for this antenna, as shown in Fig. 3a and b.

## Result analysis

**Fig. 4:**
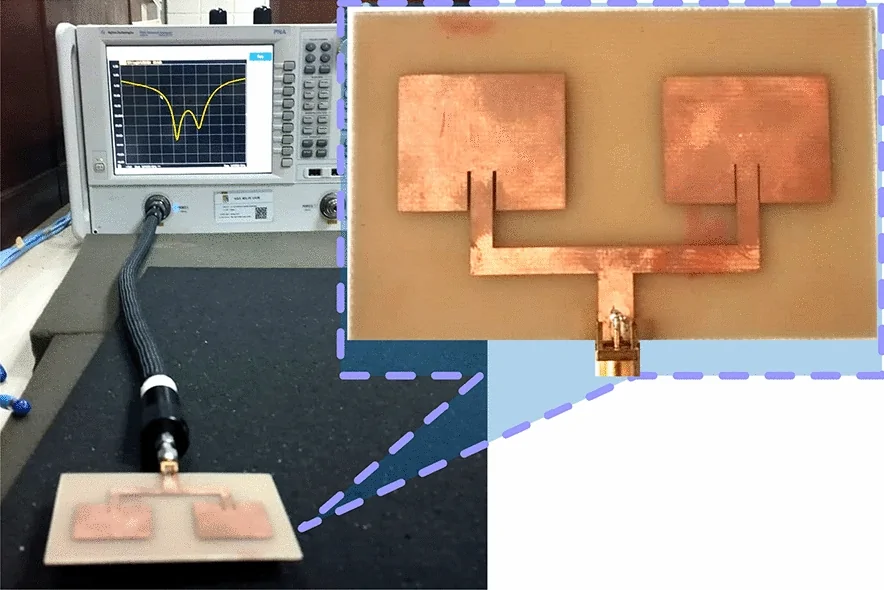
Measurement setup of the *1 × 2* array antenna.

The proposed antenna was simulated and fabricated, and the simulation results were verified through the measurements by using a vector network analyzer (VNA). Furthermore, the practical performance of the antenna was also confirmed by measuring radiation properties in an anechoic chamber. The measuring arrangement of the prototyped antenna is shown in Fig. 4.

**Fig. 5:**
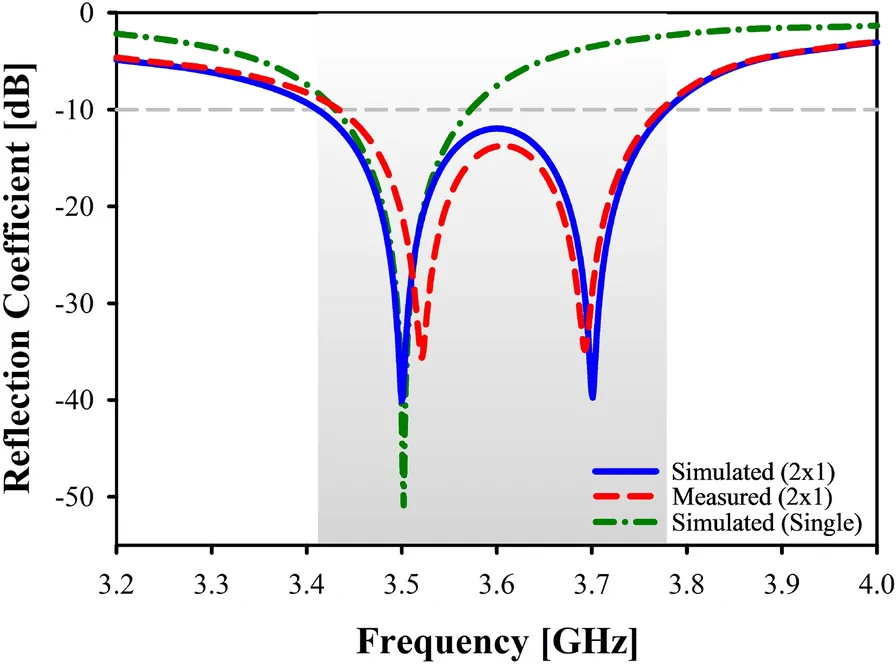
Reflection coefficient of single element and *1 × 2* array antenna.

Reflection coefficient is commonly used in the electronics and telecommunications industries to describe the amount of energy that is lost due to reflection. When evaluating the performance of transmission lines, antennas, and connections, it is frequently compared to the reflection coefficient^17^. In Fig. 5, a single antenna is contrasted with a *1× 2* array antenna. The resonance frequency of the single antenna is at only reach 3.5 GHz, which can only accommodate the bandwidth requirement of the n78 band. The *1× 2* array antenna, on the other hand, demonstrates dual applications that satisfy both the N77 and N78 bands with resonances at 3.5 GHz and 3.7 GHz. The antenna demonstrates adequate performance in both of the resonance frequencies with reflection coefficients of -40.5 dB, and -38.3 dB.

**Fig. 6:**
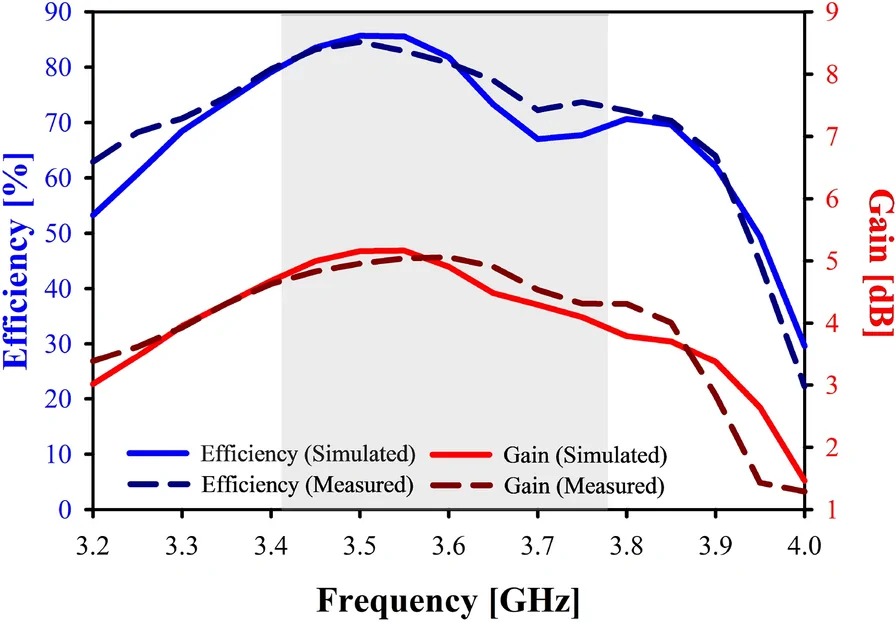
Gain and Efficiency of the *1 × 2* array antenna.

Antenna gain is a measurement of how much more electromagnetic energy an antenna can transmit or receive in one direction than a theoretical isotropic antenna. How well an antenna can send or receive electromagnetic waves in each direction is measured by this parameter^18^. The efficiency of an antenna is defined as how well it transfers electrical energy into radiated electromagnetic waves. The percentage of input power that is successfully radiated as usable electromagnetic energy is directly proportional to the efficiency of the device^19^. The Gain and Efficiency of the antenna proposed antenna is depicted in Fig. 6, where the antenna features a peak gain of 5.1 dB with maximum efficiency of 85%.

**Fig. 7:**
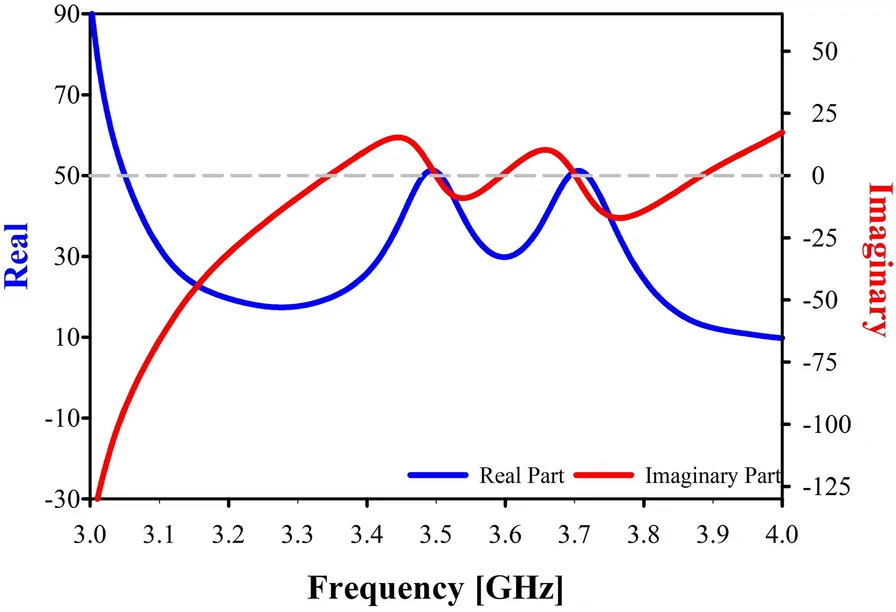
Z-parameter of the *1 × 2* array antenna.

The impedance network of an antenna system is represented mathematically by the Z-matrix, which has another name is impedance matrix^20^. Typically, 50 ohms is used for the real component (resistance) of an antenna’s impedance when discussing impedance matching of the proposed antenna. The suggested antenna has a resistive impedance and no capacitive or inductive reactance when the impedance’s imaginary part (reactance) is 0 as depicted in Fig. 7.

**Fig. 8:**
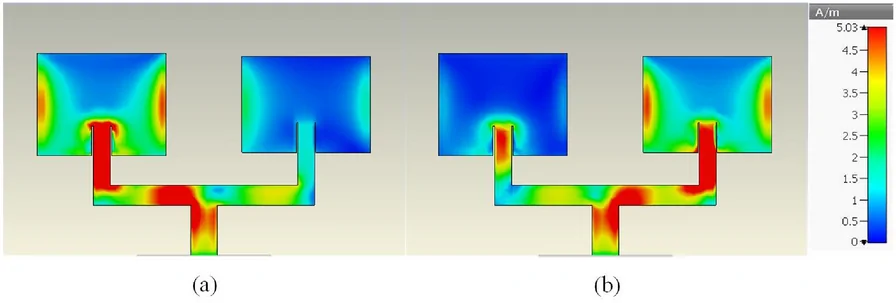
Current distribution of the *1 × 2* array antenna at (**a**) 3.5 GHz (**b**) 3.7 GHz.

Figure 8 depicts the distribution of the current at two different frequency ranges. At 3.5 GHz, the peak surface current is 109.177 A/m, whereas at 3.7 GHz, it is 198.352 A/m. The difference between these two values is due to the difference in frequency. The strength of the surface current is mirrored in the hue, which works as a visual representation of the notion. On the other hand, the left feed at 3.5 GHz and the right feed at 3.7 GHz both have a higher surface current intensity than the center feed.

**Fig. 9:**
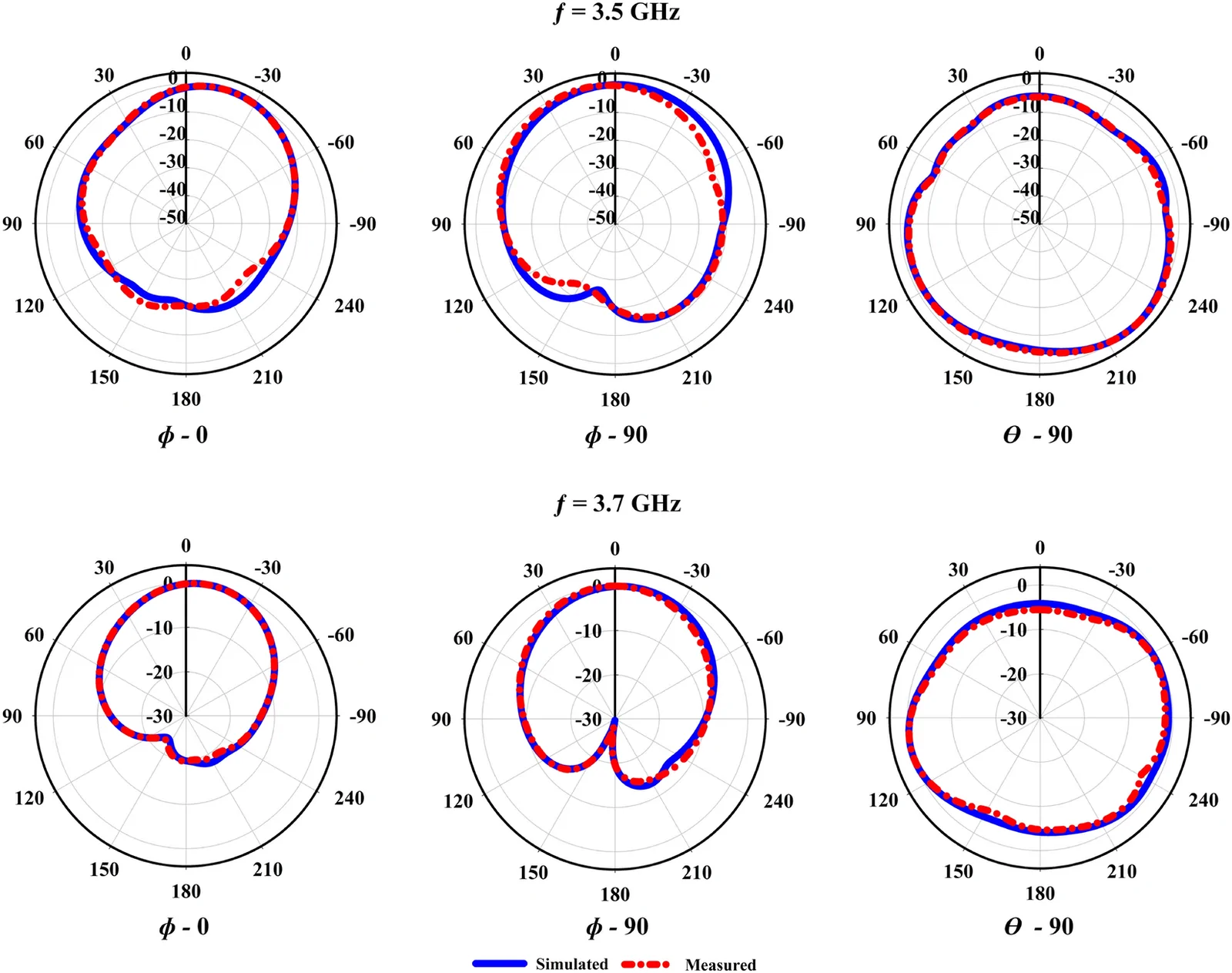
Simulaetd and measured radiation pattern of the *1 × 2* array antenna at (**a**) 3.5 GHz (**b**) 3.7 GHz.

As the radiant power in different directions, this section should show how the proposed antenna radiates in terms of its directional performance. The radiation pattern is plotted. In the polar type coordinate system, the antenna is presumed to be located at the origin, and the azimuth or horizontal angle is taken as the x-axis. Figure 9 illustrates the simulated and measured 2D radiation patterns at the frequencies of operating 3.5 GHz and 3.7 GHz. At 3.5 GHz, the maximum magnitude of the E-field pattern at *Φ* = 0$^\circ$ of the main lobe is 18.9 dBV/m and the 3 dB angular beamwidth at *Φ* = 90$^\circ$ is 83.8$^\circ$. Moreover, the E-field pattern at *θ* = 90$^\circ$ has 10.5 dBV/m main lobe magnitude. At the other resonance frequency of 3.7 GHz, the antenna gives 18.2 dBV/m for the main lobe magnitude with a 3 dB angular beamwidth of 85.4$^\circ$. In the same way, at *θ* = 90$^\circ$, the E-field characteristic reaches 9.77 dBV/m main lobe magnitude. The antenna exhibits stable radiation performance at two resonant frequencies with good beamwidth and field strength for each one, which means the antenna is suitable for the application band.

## Equivalent circuit model

An associated RLC circuit for the *1× 2* THz array antenna is established in Keysight Advanced Design System (ADS) to investigating the proposed structure is for the electromagnetic characteristics. Here, the resistor, inductor, and capacitor in this equivalent circuit represent the radiation loss, magnetic and electric energy storage , respectively. The starting values of the circuits are obtained from CST Studio Suite and then optimized in ADS using a bottom-up approach. This procedure of tuning brings the reflection coefficient response very close to the full-wave CST simulated S11 results.

The series RLC branch R1-L1-C1, which models the input transmission line and the matching impedance between the excitation source and the radiating array structure, is the principal feed network of the array antenna. To ensure equal excitation in the operating frequency band, the port impedance is changed to Z=51.5 *Ω*. The series R3-L3-C3 represents the transmission feed line to Antenna 1, and R2-L2-C2 represents the feed line to Antenna 2. These branches simulate the behaviour of signals transmitted between the radiating elements and the feed network.

The proposed array antenna can achieve dual resonant. The first resonance is established by the parallel RLC combinations R4-L4-C4 and R6-L6-C6, modelled for Ant 2 and Ant 1, respectively. The second resonance comes from the parallel R5-L5-C5 and R7-L7-C7. Such resonant branches, allowing current flow and storing electromagnetic energy in a localised manner, make it possible for dual-resonance operation. The RLC values in ADS are modified in several steps to improve resonance matching and the impedance matching to the CST simulated response.

**Fig. 10:**
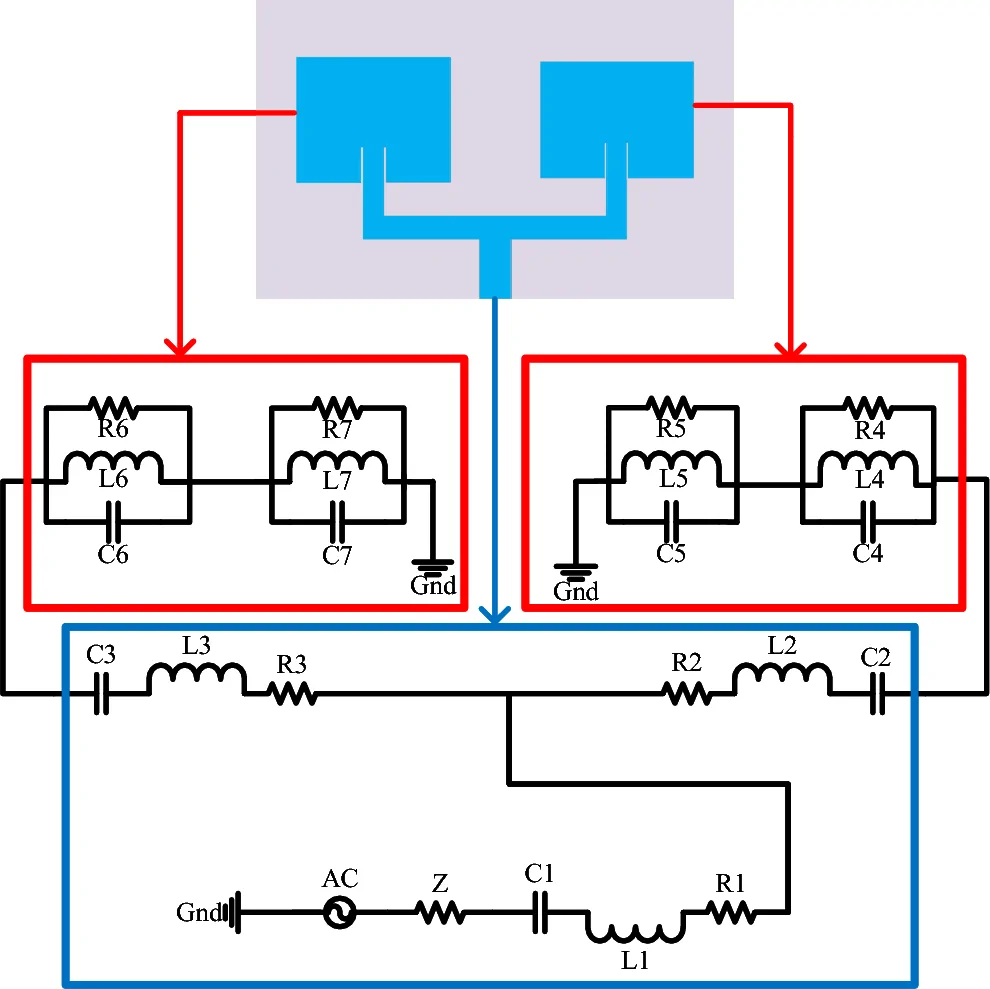
RLC representation of the *1 × 2* array antenna model.

**Fig. 11:**
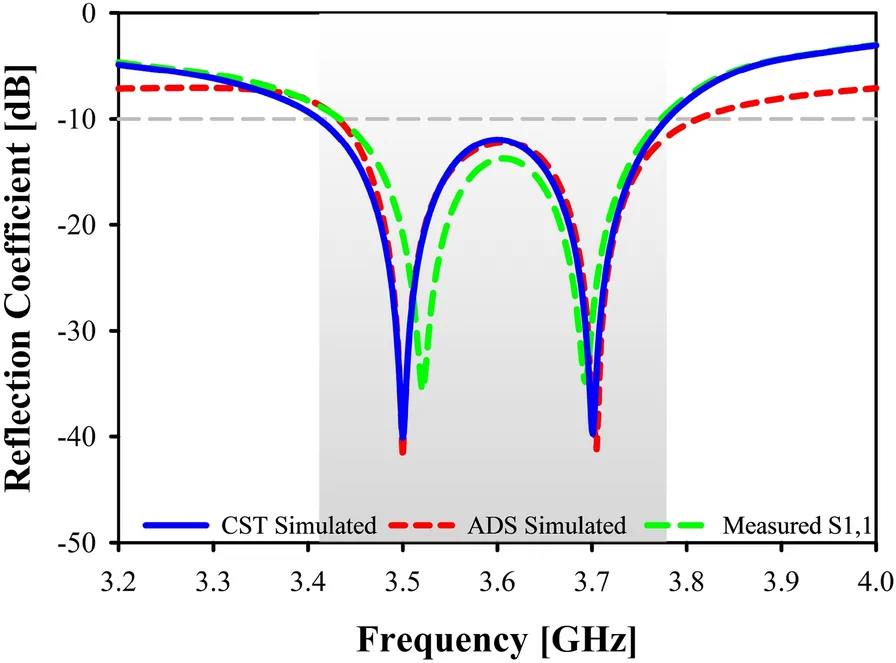
Reflection coefficient of the RLC model.

Identical RLC values are assigned to both antenna elements because of the symmetric *1× 2* array configuration, which leads to balanced EM characteristics and similar resonance performance. The resulting equivalent RLC circuit is depicted in Fig. 10, and the reflection coefficient responses derived from ADS and CST simulations are compared in Fig. 11. The extracted RLC parameter values used in the equivalent circuit are summarized in Table 1. The strong correlation between CST and ADS responses validates the accuracy and reliability of the proposed RLC equivalent circuit model.

**Table 1:** RLC values of the *1 × 2* array antenna.

Resistance	Value	Capacitance	Value	Inductance	Value
R1	7.1 *Ω*	C1	2.925 pF	L1	1.632nH
R2	12.84 *Ω*	C2	1.794941 pF	L2	0.7298485 nH
R3	34.08 *Ω*	C3	0.00005 pF	L3	2.00004 nH
R4	43.3 *Ω*	C4	0.0349 pF	L4	0.060495 nH
R5	31.1 *Ω*	C5	0.0448 pF	L5	0.041535 nH
R6	43.3 *Ω*	C6	0.0349 pF	L6	0.060495 nH
R7	31.1 *Ω*	C7	0.0448 pF	L7	0.041535 nH
Z	51.5 *Ω*				

## Curve fitting

Several regression models can be fitted using the MATLAB Curve Fitting tool, such as polynomial, exponential, Gaussian, and several other relevant curve types. In this section, the two linear segments and the best-fit regression curve are plotted for each antenna characteristic. A regression analysis is performed to analyze the change trends of the antenna parameters as a function of resonant frequency and bandwidth. The data set considered for analysis is acquired from simulations based on CST for a variety of different antenna sizes. The following subsection is devoted to the effect of changing the left and right patch lengths on the total antenna.

### Left patch length

The effect of the length of the left patch on the antenna resonance frequency and bandwidth is presented in this section.

**Fig. 12:**
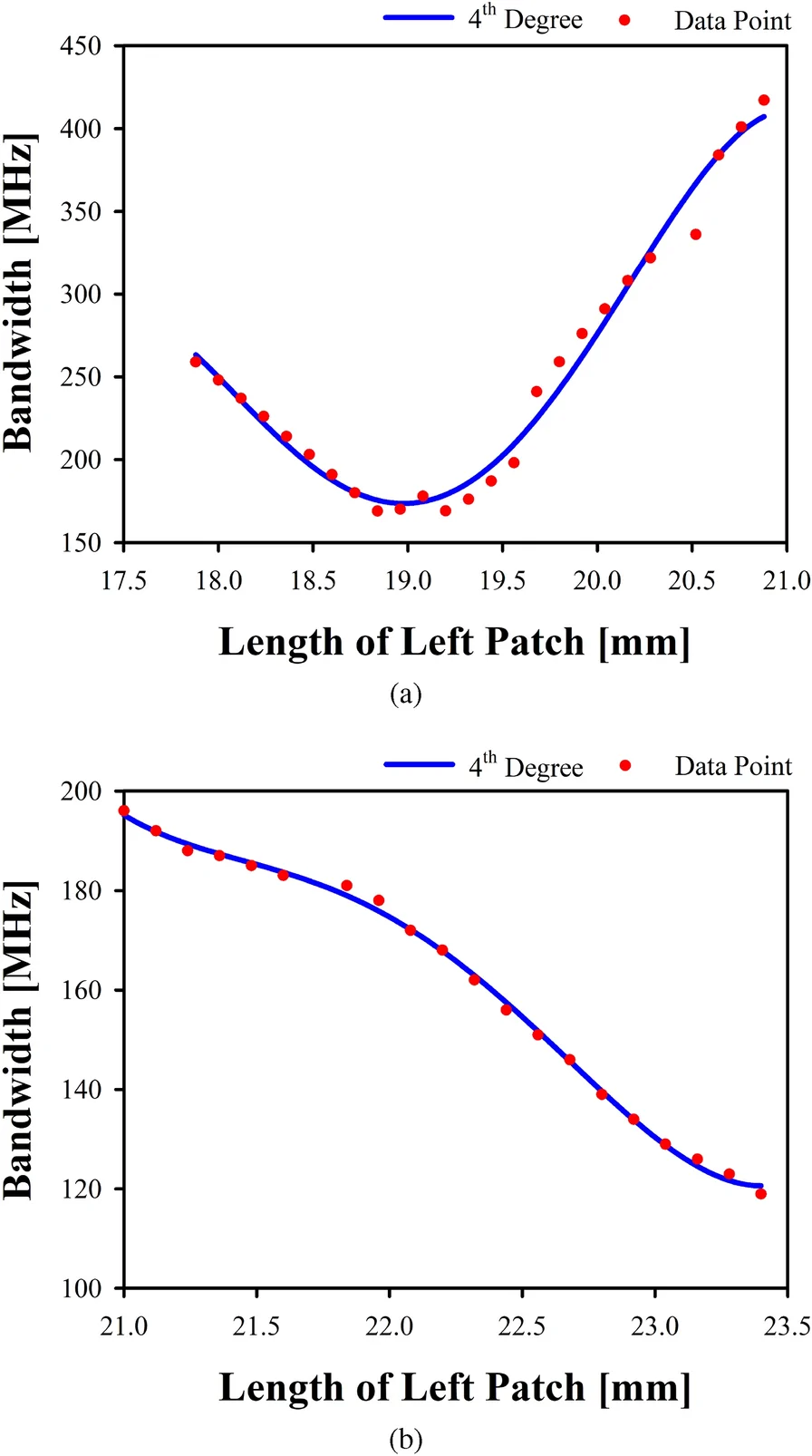
Linear regression graph to the simulated bandwidth of the antenna for left patch, between (**a**) 17.5–21 mm. (**b**) 21–23.5 mm.

The simulated bandwidth variation with respect to the length of left patch is illustrated in Fig. 12a and b. Figure 12a presents the bandwidth response ranging from 17.5 mm to 21 mm. In this region, the bandwidth initially decreases around 19 mm, after which it increases sharply. As shown in Fig. 12b, the bandwidth exhibits a monotonic decreasing trend in the range 21 mm to 23.5 mm. The linear regression curve matched with the simulated data as given in the Eqs.6 and 7.

6 $$\begin{aligned} -0.02 x^4 +1.25 x^3 -35.71 x^2 +452.62 x -2145.02 \end{aligned}$$

7 $$\begin{aligned} 0.01 x^4 -0.84 x^3 +27.64 x^2 -405.97 x +2234.69 \end{aligned}$$

**Fig. 13:**
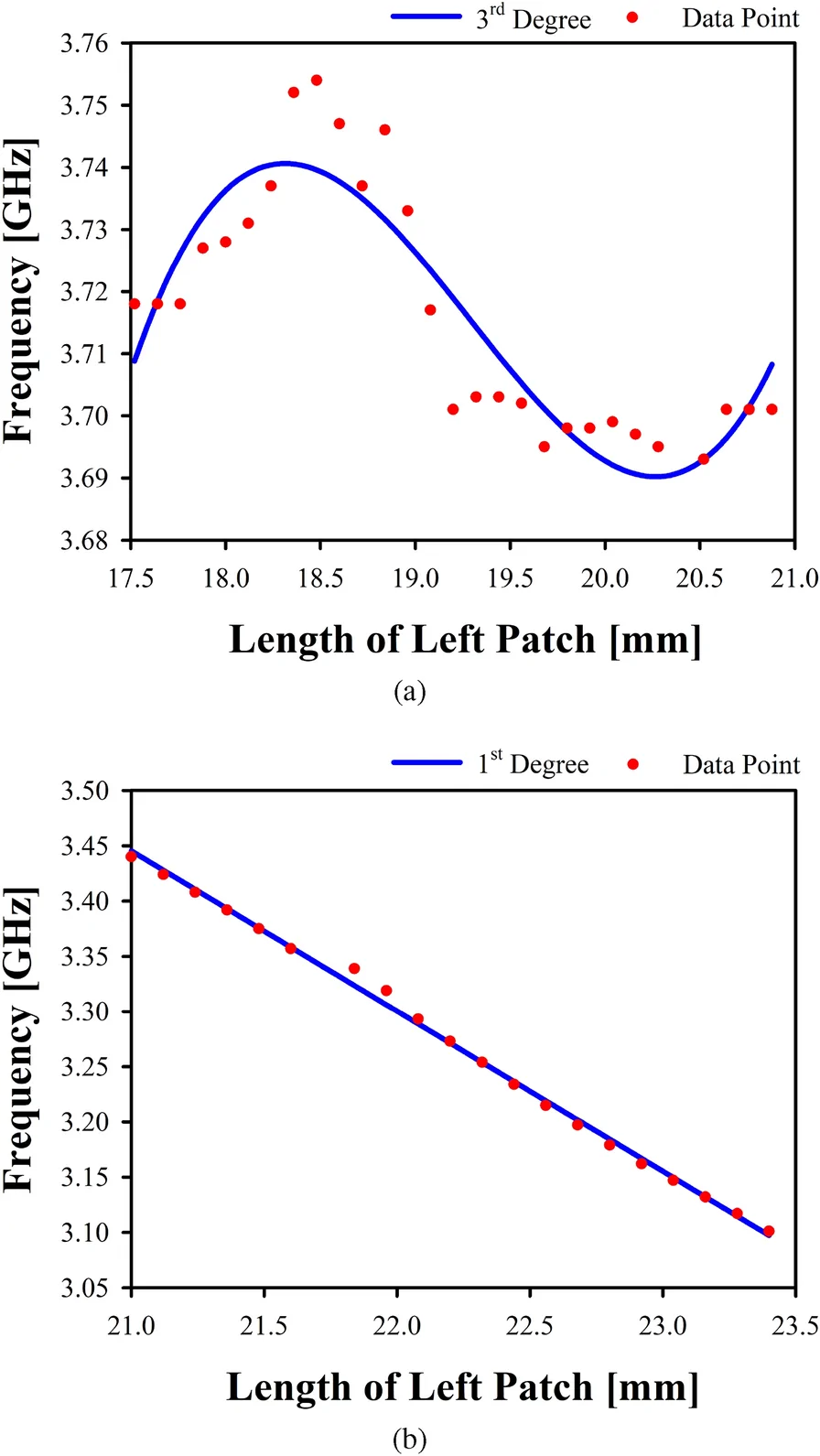
Linear regression graph to the simulated frequency of the antenna for left patch, between (**a**) 17.5–21 mm. (**b**) 21–23.5 mm.

The simulated resonant frequency variation with respect to the left patch length is divided into two regions and illustrated in Fig. 13a and b, respectively. Figure 13a shows a nonlinear variation of the resonant frequency as length increases from 17.5 mm to 21 mm. Figure13b illustrates a steady decreasing trend in resonant frequency for length varying from 21 mm to 23.5 mm. The linear regression curves are given in the Eqs. 8 and 9.

8 $$\begin{aligned} 0.01 x^3 -0.78 x^2 +15.10 x -92.88 \end{aligned}$$

9 $$\begin{aligned} -0.15 x +6.49 \end{aligned}$$

### Right patch length

The effect of the suitable patch size on the antenna bandwidth and resonance frequency is described in this section.

**Fig. 14:**
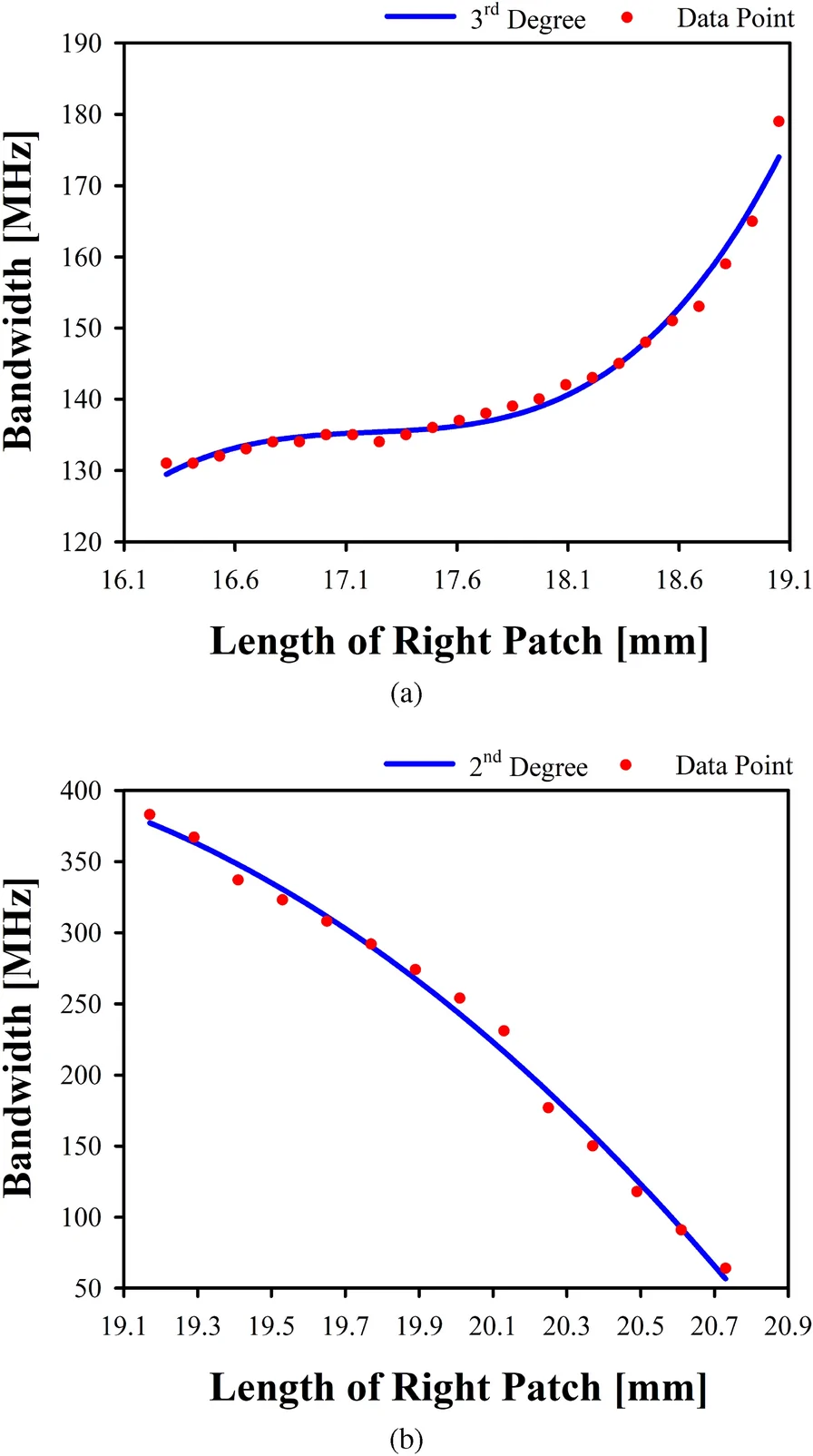
Linear regression graph to the simulated bandwidth of the antenna for right patch, between (**a**) 16.1–19.1 mm. (**b**) 19.1–20.9 mm.

The simulated bandwidth variation with respect to the length of right patch is divided into two regions and illustrated in Fig. 14a and b, respectively. Figure 14a illustrates a monotonically increasing trend in bandwidth frequency for length varying from 16.1 mm to 19.1 mm. Whereas the bandwidth decreases with increasing length in range from 19.1 mm to 20.9 mm, as shown in Fig. 14b. The linear regression curve matched with the simulated data as given in the Eqs. 10 and 11.

10 $$\begin{aligned} 0.01 x^3 -0.30 x^2 +5.20 x -29.75 \end{aligned}$$

11 $$\begin{aligned} -0.06 x^2 +2.32 x -20.87 \end{aligned}$$

**Fig. 15:**
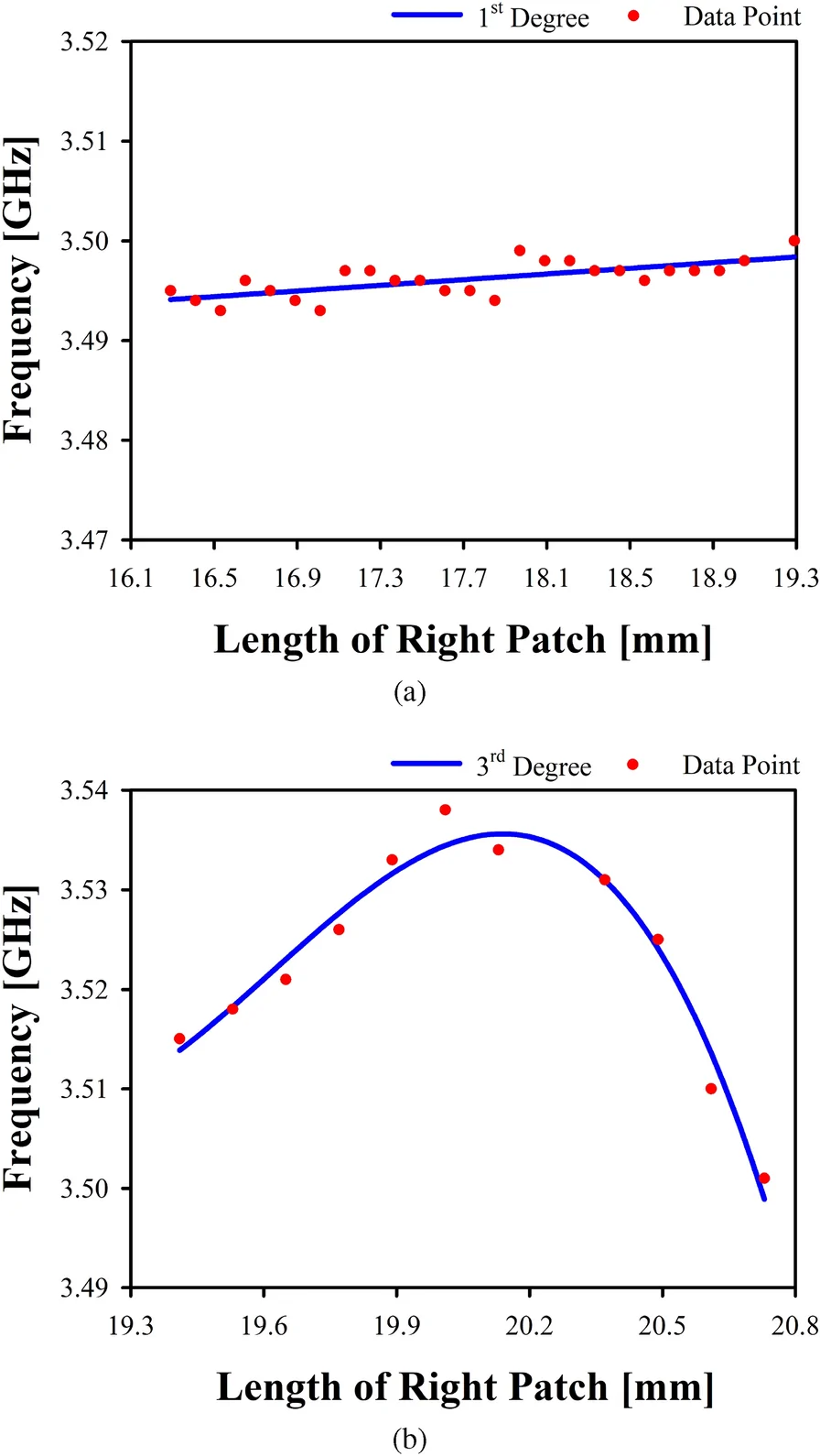
Linear regression graph to the simulated frequency of the antenna for right patch, between (**a**) 16.1–19.3 mm. (**b**) 19.3–20.8 mm.

The simulated resonant frequency variation with respect to the length of the right patch is illustrated in Fig. 15a and b. As shown in Fig. 15a, the resonant frequency exhibits a gradual increasing trend as the right patch length varies from 16.1 mm to 19.3 mm. Whereas Fig. 15b presents the resonant frequency behavior for a higher range of the right patch length, from 19.3 mm to 20.8 mm. In this region, the frequency initially increases and then decreases with further increase in length. The linear regression curve matched with the simulated data as given in the Eqs. 12 and 13.

12 $$\begin{aligned} 0.001 x +3.471 \end{aligned}$$

13 $$\begin{aligned} -0.05 x^3 +2.86 x^2 -56.16 x +370.19 \end{aligned}$$

## Machine learning analysis

Machine learning models can be very helpful in antenna validation^21^. Regression-based machine learning has performed very well in predicting antenna parameters as regression can establish effects and relations between variables. In this study, the CST studio suite has been utilized to collect the data by changing the antenna parameter. Varying the length and width of the patch, feed line, and changing distance of two elements; the results of the antenna have been recorded. The data is used for training different regression model and validating their performance. The use of multiple regression models is very important for achieving optimal results^22^.

**Fig. 16:**
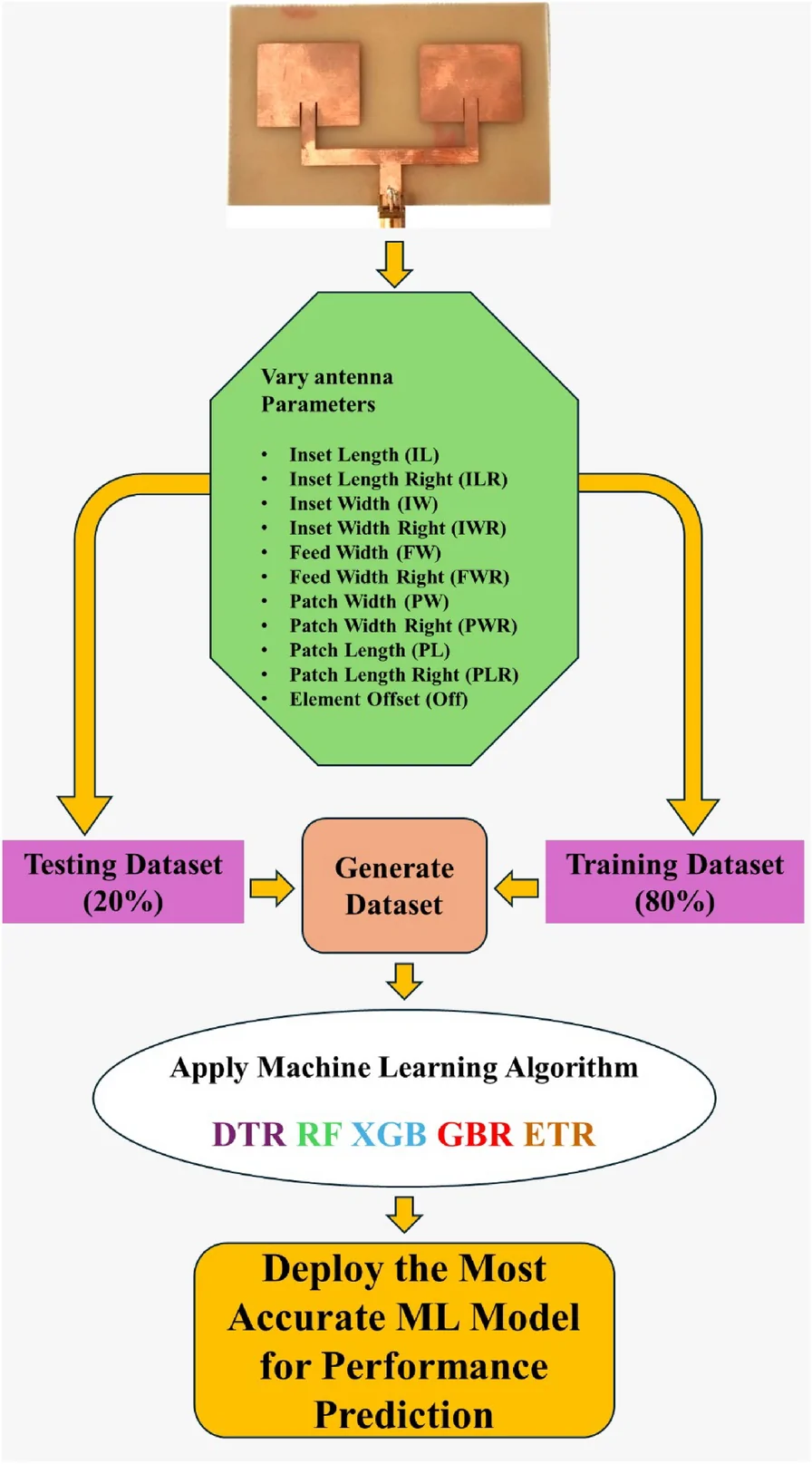
Machine Learning Methodology.

*Step* 1: Antenna Design and Parameter Selection

In the first step, the proposed antenna structure was selected as the base model for the study. The important geometrical parameters of the antenna were identified, including inset length, inset width, feed width, patch width, patch length, right-side dimensions, and element offset. These parameters were selected because they strongly influence the antenna’s performance (Fig. 16).

*Step* 2: Variation of Antenna Parameters

After selecting the important antenna parameters, their values were varied within suitable ranges. Different combinations of these parameters were used to create multiple antenna design samples. This step was necessary to observe how changes in antenna dimensions affect the antenna performance.

*Step* 3: Dataset Generation

In order to obtain the required performance values, a simulation was run for each antenna configuration. The corresponding antenna performance values were considered as output or target data, while the antenna parameters were considered as the input features. Thus, a full data set is generated for machine prediction based on learning.

*Step* 4: Dataset Splitting

The synthesized data was split into training and test sets. In particular, 80% of the data were used for training the machine learning models, and the rest 20% was used for testing. Such data splitting allows the models to be trained on the training samples and then tested for their predictive power on novel data.

*Step* 5: Machine Learning Model Development

Machine Learning Model Development The dataset was processed and tested through multiple machine learning regression algorithms such as Decision Tree Regressor, Random Forest Regressor, XGBoost Regressor, Gradient Boosting Regressor, and Extra Trees Regressor. The training models, which were based on various parameter combinations of antenna design and performance, were utilized to reproduce the design parameters from the given performance characteristics.

*Step* 6: Model Evaluation and Deployment

After training, the models were tested using the testing dataset. Their predicted results were compared with the simulation results using performance metrics such as MAE, MSE, RMSE, and R$^2$. Finally, the model with the highest accuracy and lowest prediction error was selected and deployed for antenna performance prediction.

In this study, five regression machine learning models (Decision Tree Regression, Extra Trees Regression, Random Forest Regression, Gradient Boosting Regression, and XGB Regression) were used for training.

*Decision tree regression* Decision tree regression is a type of supervised learning algorithm that is used when we want to predict continuous values. It builds a tree-like structure by separating the data set into smaller partitions along a series of decision rules and finds the parameters of antenna performance efficiently^23^.

*Random Forest Regression* The random forest regression is an ensemble-based algorithm that constructs numerous decision trees in parallel, considering different subsets of features, and averages the predictions of each tree to improve performance^24^. For antenna design applications, this is a very powerful technique, as it allows for processing of high-dimensional input space and capturing complex nonlinear mappings between design variables and performance metrics.

*XGB Regression* XGB Regression, derived from the XGBoost framework, leverages the optimized boosting algorithms for enhanced speed and prediction accuracy. It offers regularization, parallel processing, and good handling of missing data. Thus, it can be utilized to estimate antenna behaviors such as gain, directivity, radiation pattern, bandwidth, and resonant frequency from simulated / measured data sets^25^.

*Gradient Boosting Regression* Gradient boosting Regression is an ensemble learning method that builds models sequentially. Each new model attempts to approximately unlearn the errors made by the previous model and reduce the prediction error. It is a powerful tool for creating reliable and precise regression models^26^, because it can describe complicated nonlinear influences.

*Extra Tree Regression* To generate a large number of decision trees, the Extra Tree Regression ensemble method uses a stochastic procedure to select feature subsets and thresholds. The high level of randomisation greatly improves the robustness and efficiency of the model. Based on a voting scheme on the predictions, the Extra Trees regressor is also a good technique for regression problems, as it reduces the risk of overfitting^27^.

To get the best-performed model, the performance of the models needs to be compared. The performance metrics that can determine the best-performed model are MAE, MSE, RMSE, MAPE, R-squared, and Variance score.

The average variation between the computed and observed values is determined by the mean absolute error, or MAE^28^. The MAE is computed using Eq. 14.

14 $$\begin{aligned} M A E=\frac{1}{n} \sum _{i=1}^n\left| y_i-\hat{y}_i\right| \end{aligned}$$

The mean squared error (MSE) is the most commonly used form of the regression loss function. The loss is calculated as the squared differences of actual and predicted values multiplied by the mean of all data points. Equation shows the MSE formulation^29^, Eq. 15.

15 $$\begin{aligned} M S E=\frac{1}{n} \sum _{i=1}^n\left( y_i-\hat{y}_i\right) ^2 \end{aligned}$$

By taking the Root of MSE, the Root Mean Squared Error, or RMSE, returns the unit to its initial value. Eq. 16 displays the RMSE^30^ equation.

16 $$\begin{aligned} R M S E=\sqrt{\frac{1}{n} \sum _{i=1}^n\left( y_i-\hat{y}_i\right) ^2} \end{aligned}$$

The difference between the actual and anticipated values can be found first, and then divided by the actual value to get the mean absolute percentage error, or MAPE. Eq. 17 presents the formula for MAPE^31^.

17 $$\begin{aligned} M A P E=\frac{100 \%}{n} \sum \left| \frac{y_i-\hat{y}_i}{y_i}\right| \end{aligned}$$

The precision of the model fit is shown by the R-squared value. A near value of 1 for R$^2$ suggests that the model fits the data well; a value closer to 0 suggests that the model is not particularly accurate. R-squared may be negative if a model forecasts an irrational result. Eq. 18 expresses R-squared^32^.

18 $$\begin{aligned} R^2=1-\frac{\sum _{i=1}^N\left( y_i-\hat{y}_i\right) ^2}{\sum _{i=1}^N\left( y_i-\hat{y}_i\right) ^2} \end{aligned}$$

The error dispersion in every dataset is described by the explained variance score^33^. it is defined according to Eq. 19.

19 $$\begin{aligned} explained~varience(y, \hat{y})=1-\frac{Var(y-\hat{y})}{Var(y)} \end{aligned}$$

**Fig. 17:**
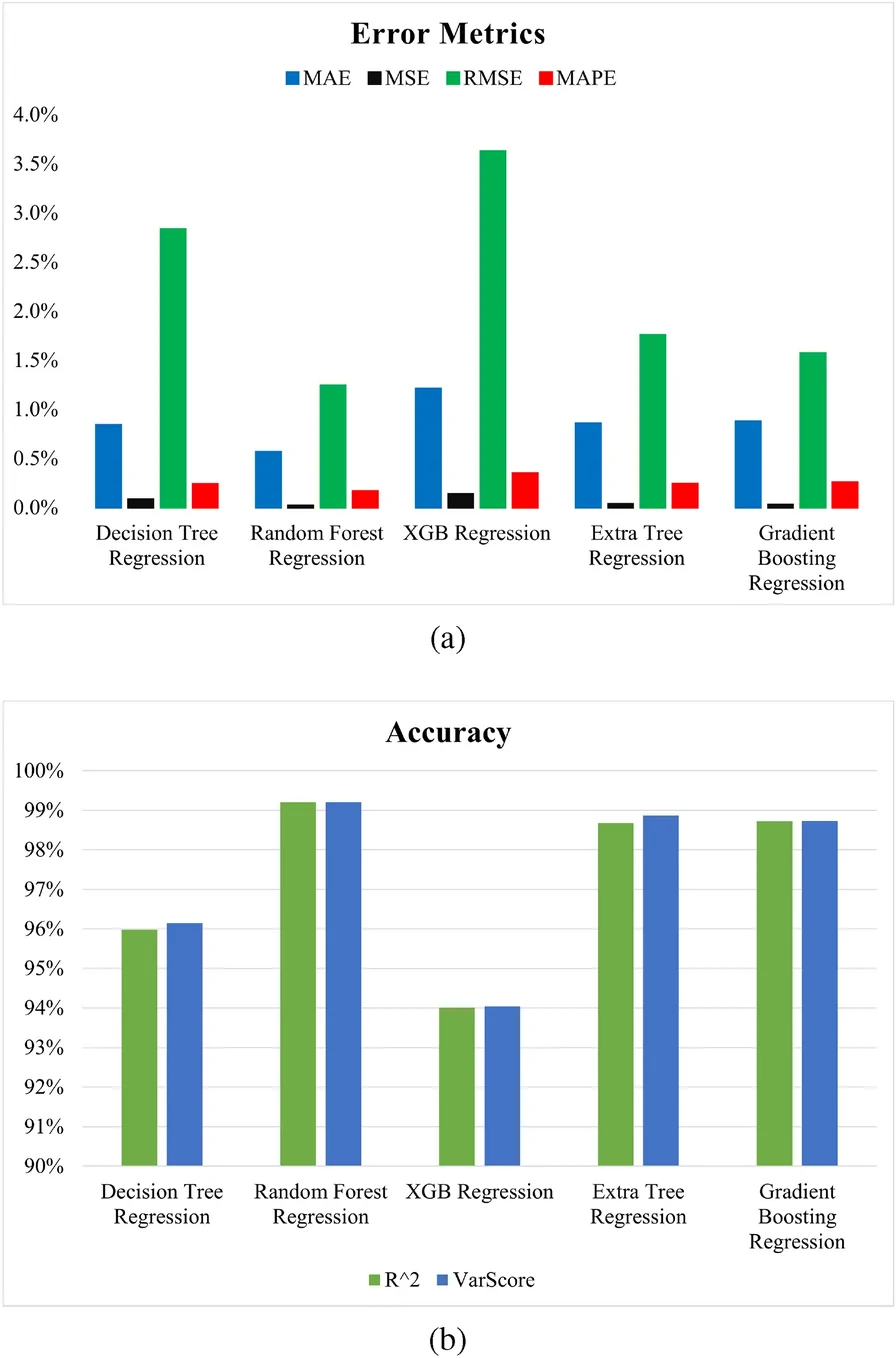
Performance comparative bar chart of ML regressors (Resonance). (**a**) Error Metrics. (**b**) Accuracy.

**Fig. 18:**
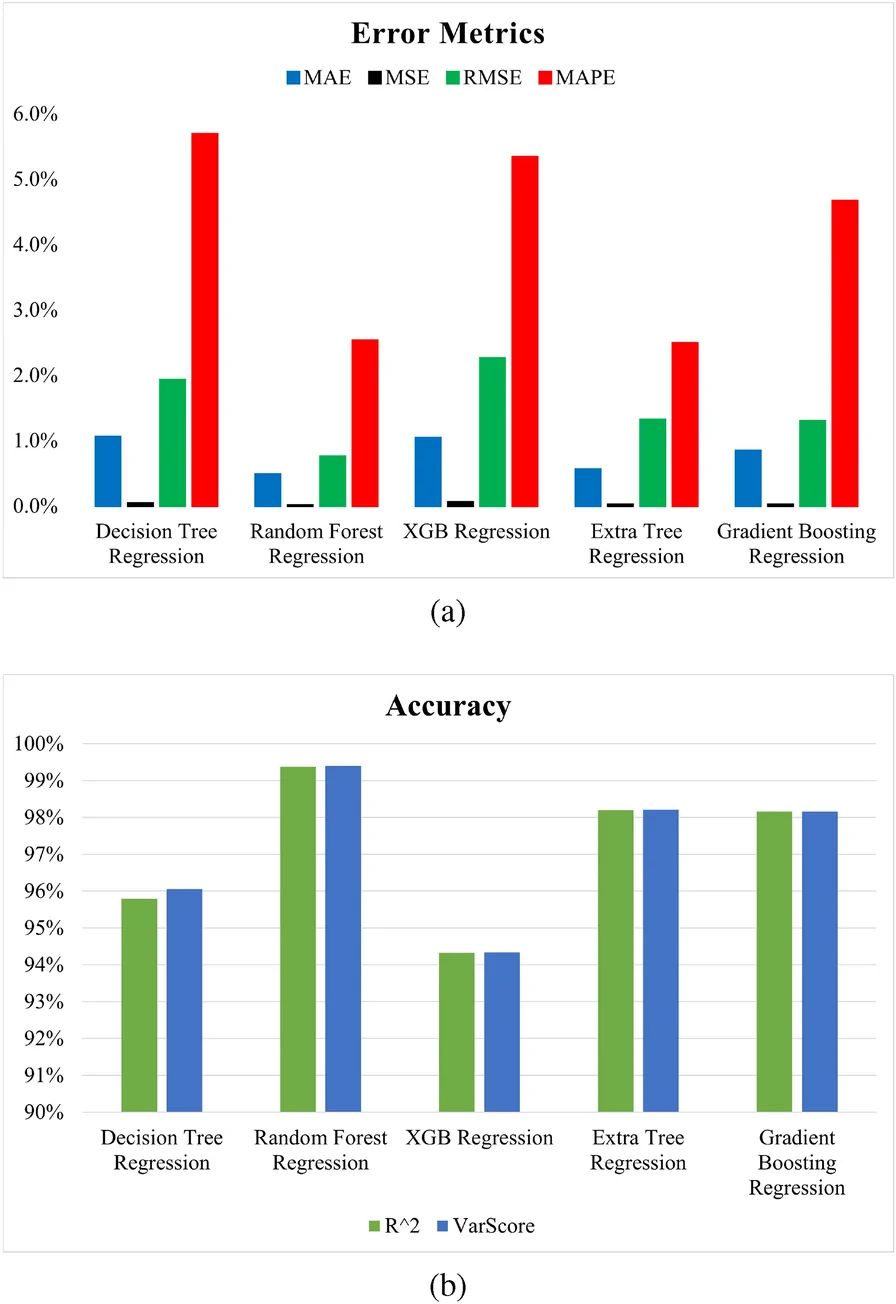
Performance comparative bar chart of ML regressors (Bandwidth). (**a**) Error Metrics. (**b**) Accuracy.

**Fig. 19:**
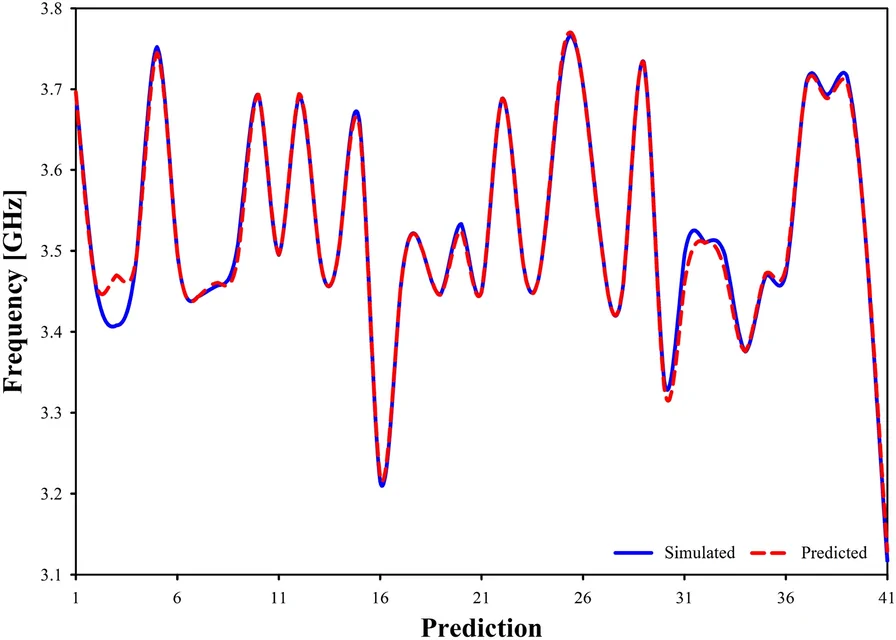
Simulated vs predicted resonant frequency using Random Forest Regression.

**Fig. 20:**
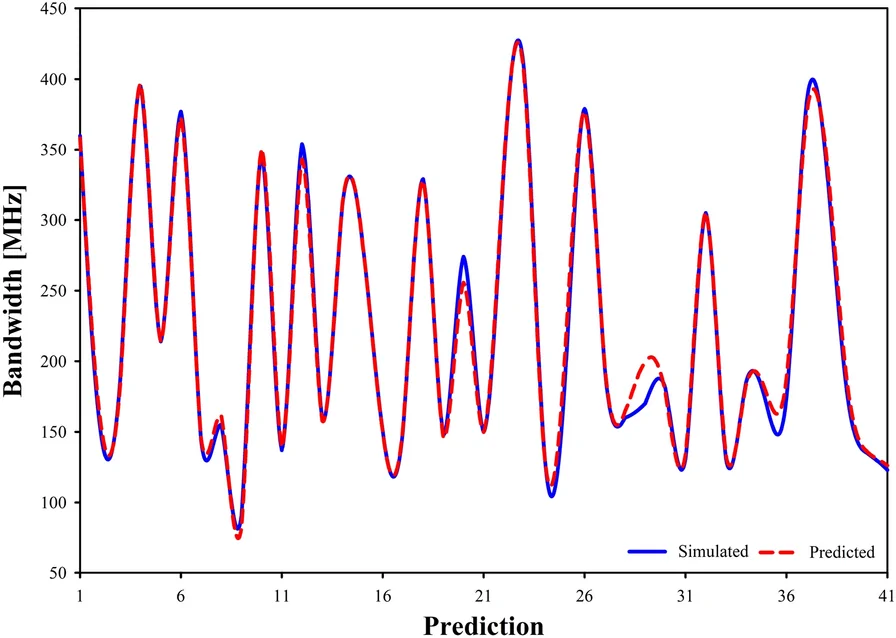
Simulated vs predicted bandwidth using Random Forest Regression.

Figures 17 and 18 illustrate the accuracy of the various tested models, and Figs. 19 and 20 illustrate the prediction errors for the resonance frequency, and the bandwidth of the antenna, respectively. The Random Forest Regression exhibited exceptional performance for predicting both frequency and bandwidth among the five regression models that were analysed. As compared to other models, the random forest regression’s prediction errors were found to be very low, leading to an amazing level of model accuracy, as proven by variance score and R-Squared as high as 99%, presented in Tables 2 and 3. Tables 4 and 5, presenting predicted and simulated values for bandwidth and frequency respectively, further support this statement. There are very small differences between the simulated and predicted values, as can be observed by analysing the tables.

**Table 2:** Performance metrics of ML regressors (Resonance).

Algorithms	MAE (%)	MSE (%)	RMSE (%)	MAPE (%)	R$^2$ (%)	VarScore (%)
Decision Tree Regression	0.8366	0.0801	2.8309	0.2339	95.9804	96.1424
Random Forest Regression	0.5621	0.0154	1.2402	0.1619	99.1979	99.1980
XGB Regression	1.2033	0.1312	3.6219	0.3461	94.0047	94.0404
Extra Tree Regression	0.8513	0.0307	1.7518	0.2374	98.6778	98.8698
Gradient Boosting Regression	0.8715	0.0245	1.5657	0.2510	98.7218	98.7302

**Table 3:** Performance metrics of ML regressors (Resonance).

Algorithms	MAE (%)	MSE (%)	RMSE (%)	MAPE (%)	R$^2$ (%)	VarScore (%)
Decision Tree Regression	1.0537	0.0370	1.9235	5.6844	95.7907	96.0507
Random Forest Regression	0.4801	0.0057	0.7540	2.5273	99.3786	99.3987
XGB Regression	1.0392	0.0507	2.2524	5.3338	94.3285	94.3363
Extra Tree Regression	0.5554	0.0173	1.3162	2.4848	98.2013	98.2048
Gradient Boosting Regression	0.8416	0.0168	1.2977	4.6630	98.1590	98.1594

**Table 4:** Simulated and predicted resonant frequency comparison on the test set Random Forest Regression.

Simulaetd	Predicted	Simulated	Predicted	Simulated	Predicted
3.6960	3.6967	3.6560	3.6491	3.7330	3.7324
3.4540	3.4574	3.2150	3.2203	3.3390	3.3317
3.4080	3.4700	3.4520	3.4521	3.4950	3.4595
3.4950	3.4955	3.5060	3.5061	3.5100	3.5097
3.7520	3.7450	3.4470	3.4458	3.4940	3.4755
3.4980	3.4977	3.5330	3.5250	3.3750	3.3755
3.4430	3.4428	3.4490	3.4497	3.4690	3.4719
3.4570	3.4609	3.6880	3.6878	3.4720	3.4787
3.5100	3.4945	3.4940	3.4940	3.7070	3.7049
3.6930	3.6932	3.4960	3.4961	3.6930	3.6885
3.4950	3.4955	3.7370	3.7436	3.7170	3.7094
3.6910	3.6939	3.7050	3.7045	3.4930	3.4960
3.4970	3.4967	3.4900	3.4894	3.1170	3.1290
3.5090	3.5087	3.4620	3.4630		

**Table 5:** Simulated and predicted bandwidth comparison on the test set Random Forest Regression.

Simulaetd	Predicted	Simulated	Predicted	Simulated	Predicted
0.3600	0.3581	0.2830	0.2830	0.1700	0.1987
0.1530	0.1594	0.1510	0.1502	0.1810	0.1783
0.1880	0.1848	0.1510	0.1527	0.1310	0.1340
0.3950	0.3967	0.3290	0.3279	0.3050	0.3056
0.2140	0.2140	0.1480	0.1460	0.1310	0.1338
0.3770	0.3728	0.2740	0.2557	0.1850	0.1830
0.1440	0.1455	0.1500	0.1498	0.1700	0.1778
0.1550	0.1615	0.3370	0.3387	0.1740	0.1878
0.0910	0.0826	0.4060	0.4032	0.3840	0.3750
0.3470	0.3506	0.1360	0.1359	0.3360	0.3429
0.1370	0.1392	0.1800	0.1976	0.1780	0.1862
0.3540	0.3431	0.3790	0.3771	0.1350	0.1360
0.1590	0.1578	0.1950	0.1972	0.1230	0.1261
0.3110	0.3119	0.1600	0.1645		

## Comparisons with the recent state of the art

**Table 6:** Performance comparisons with the recent state of the art.

Ref	Operating Frequency (GHz)	Reflection Coefficient (dB)	Bandwidth (GHz)	Peak Gain (dB)	Radiation Efficiency (%)	Board size	Config	RLC	ML Analysis
Proposed	3.5, 3.7	−40.5, −38.3	0.4	5.1	85	0.58$\lambda _\circ \times$0.93$\lambda _\circ$	*1× 2*	YES	YES
^34^	2.45, 5.4	−25, −24	0.85, 0.8	4.25, 5.4	95	0.15$\lambda _\circ \times$0.26$\lambda _\circ$	*1× 2*	NO	NO
^35^	2.4	−55	1.2	−5.1	–	0.58$\lambda _\circ \times$0.35$\lambda _\circ$	*1× 2*	NO	NO
^12^	2.4	−27	0.11	1.58	–	0.96$\lambda _\circ \times$0.88$\lambda _\circ$	*1× 2*	NO	NO
^13^	5.8	−24	0.14	3.55	65	–	*1× 2*	NO	NO
^14^	3	−45	3.79	0.53	41	0.66$\lambda _\circ \times$0.45$\lambda _\circ$	*2× 2*	NO	NO
^15^	2.45	−30	0.49	3.56	76	0.78$\lambda _\circ \times$0.86$\lambda _\circ$	*2× 2*	NO	NO
^36^	2.3, 2.4, 3.5	−23.7, −22.87, −20.60	0.24, 0.21	4.69, 8.53, 3.49	–	–	*1× 3*	NO	NO
^37^	3.05–6.32	−20	3.28	3.07	–	0.31$\lambda _\circ \times$0.31$\lambda _\circ$	*1× 2*	YES	NO

Table 6 compares the proposed antenna with previously reported array antennas in terms of operating frequency, reflection coefficient, gain, radiation efficiency, substrate, structural complexity, and analytical capability. The proposed two-element FR4-based array operates at 3.5 and 3.7 GHz, making it suitable for sub-6 GHz wireless applications. It achieves strong impedance matching, with reflection coefficients of -40.5 and -38.3 dB, which are significantly better than those reported in^12,13,15,34,36,37^. Although^35^ and^14^ exhibit deeper reflection coefficients of -55 dB and -45 dB, respectively, their reported gain and efficiency are considerably lower than those of the proposed design. The proposed antenna provides a peak gain of 5.1 dB, outperforming the designs in^12–15,35^ and^37^, whose gains range from -5.1 to 3.56 dB, except for selected operating bands in^34^ and^36^. In addition, the proposed design achieves a radiation efficiency of 85%, which is higher than the efficiencies reported in^13–15^, namely 65%, 41%, and 76%, respectively. Although^34^ reports a higher efficiency of 95%, the proposed work offers improved reflection performance together with additional analytical features. From an implementation perspective, the proposed antenna is fabricated on low-cost FR4 substrate and uses only a two-element array, while several existing designs employ four-element structures or larger board dimensions. Most importantly, the proposed work uniquely incorporates both RLC equivalent-circuit analysis and machine-learning-based analysis. Among the compared studies, only^37^ includes RLC analysis, while none of the reference designs reports the integration of machine learning. Therefore, the proposed design provides a balanced improvement in impedance matching, gain, radiation efficiency, structural simplicity, and intelligent performance analysis, demonstrating its potential for compact sub-6 GHz array antenna applications.

## Conclusions

In this work, the antenna under evaluation is the subject of a simulation, experimental measurement, supervised regression- based machine learning, and an RLC equivalent circuit model. The proposed design is very compact, and the performance is good; the single-stage conversion efficiency is 85%. To verify the expected behaviour of the antenna, a prototype was designed, fabricated, and measured. The resonant frequencies generated by using CST simulation and measurement are in good agreement with those predicted by the RLC equivalent model via ADS Agilent. In addition, 5 supervised regression machine learning models were executed to predict the bandwidth and resonant frequency of the presented antenna. The predicted results are in good agreement with the simulated values, which ensures the reliability of the machine learning technique. For future work, a more comprehensive dataset would be collected to predict the operating frequencies of multiband antennas using deep learning models or the integration of them, such as ANN and CNN. More improvement will be focused on the better impedance matching of the proposed MPA with the SMA connector to minimize the deviation between the simulated and measured resonant frequencies. In general, the simulated and measured, circuit modeled, and predicted results verify that the N77/N78 array antenna performance is strong and applicable.

## Data Availability

The datasets generated and/or analyzed during the current study are available from the corresponding author upon reasonable request.
